# Supplementary material for: Household food insecurity is associated with greater autonomic dysfunction testing score in Latinos with type 2 diabetes
Source: PLoS One. 2024 Feb 23;19(2):e0297681. doi: 10.1371/journal.pone.0297681 (PMC10889858; doi:10.1371/journal.pone.0297681)
Supplement: Attachment — Submitted filename: Reviewers comment.docx [file pone.0297681.s001.docx]

**Reviewer’s comments**

The study investigated the nexus between household food insecurity (HFI) and autonomic nervous system (ANS) function in a subset of low-income Latinos with type 2 diabetes with data from a stress management trial. Generally, the article is well-written. However, the authors need to improve it. Below are my suggestions and comments.

**Abstract**

Methods: Present the methods adopted in sentences; avoid the use of symbol where necessary for easy understanding by the readers.

Results: Present the results in sentences; avoid the use of symbol where necessary for easy understanding by the multidisciplinary readers.

**Introduction**

The introduction is well-written. However, the authors can improve this section by clearly showing the research gap and how their study contributes to literature. In addition, they should include how the study is important to justify the current study.

Paragraph 2: define FI in the first use.

**Results**

The sentence “*A total of 121 individuals had baseline assessments for the PARENT STUDY trial, 182 n=107 were randomized and n=96 completed post assessments. Of them, n = 11 did not wear 183 a Holter monitor, HRV data from n = 15 required >20% .20% interpolation and so were 184 excluded, and 1 individual did not provide food security data. Of the remaining 69, n=34 185 declined the substudy or were excluded due to hand dysfunction (e.g., arthritis) that precluded 186 the handgrip test. This yielded 35 participants for analysis*” would be better under methods.

**Discussion**

The authors need to further discuss their findings by clearly given implications of each finding and link them with earlier studies that are in line or against their findings.

**Conclusion**

The authors need to have few sentences to conclude the paper in addition to what they have in this section. What the authors currently have under this section is recommendation, which should come after drawing inferences from the study.
