# Supplementary material for: Household food insecurity is associated with greater autonomic dysfunction testing score in Latinos with type 2 diabetes
Source: PLoS One. 2024 Feb 23;19(2):e0297681. doi: 10.1371/journal.pone.0297681 (PMC10889858; doi:10.1371/journal.pone.0297681)
Supplement: Attachment — Submitted filename: Response to reviewers 11.18.23.docx [file pone.0297681.s002.docx]

To: Olutosin Ademola Otekunrin, PhD, Academic Editor, PLOS ONE

From: Julie Wagner

Date: 11/4/23

Re: PONE-D-23-25306 “Household food insecurity is associated with greater autonomic dysfunction testing score in Latinos with type 2 diabetes”

We thank the editor and reviewers for their careful reviews. We were pleased with their overall positive impression of the manuscript. Below we respond to comments in **bold font** for easy spotting. We have made all the changes suggested by the reviewers. With their help, we have fashioned an even stronger manuscript.

Comments from the Editor:

1. Please ensure that your manuscript meets PLOS ONE's style requirements, including those for file naming. The PLOS ONE style templates can be found at

[https://journals.plos.org/plosone/s/file?id=wjVg/PLOSOne_formatting_sample_main_body.pdf](https://urldefense.com/v3/__https:/journals.plos.org/plosone/s/file?id=wjVg*PLOSOne_formatting_sample_main_body.pdf__;Lw!!Cn_UX_p3!nLZ4s0aPjA7pGdWxOgrjTLXNHw7jk61rge1vF7GbZjIYvd6yZp9vi0UsH5KkbK-KftW5u4tH6MTIxodbeQ$) and

[https://journals.plos.org/plosone/s/file?id=ba62/PLOSOne_formatting_sample_title_authors_affiliations.pdf](https://urldefense.com/v3/__https:/journals.plos.org/plosone/s/file?id=ba62*PLOSOne_formatting_sample_title_authors_affiliations.pdf__;Lw!!Cn_UX_p3!nLZ4s0aPjA7pGdWxOgrjTLXNHw7jk61rge1vF7GbZjIYvd6yZp9vi0UsH5KkbK-KftW5u4tH6MQFIDXfiw$)

2. We noticed you have some minor occurrence of overlapping text with the following previous publication(s), which needs to be addressed:

- [https://doi.org/10.2337/db19-2284-PUB](https://urldefense.com/v3/__https:/doi.org/10.2337/db19-2284-PUB__;!!Cn_UX_p3!nLZ4s0aPjA7pGdWxOgrjTLXNHw7jk61rge1vF7GbZjIYvd6yZp9vi0UsH5KkbK-KftW5u4tH6MS3SN0QYg$)

- [https://doi.org/10.1093/cdn/nzaa043_013](https://urldefense.com/v3/__https:/doi.org/10.1093/cdn/nzaa043_013__;!!Cn_UX_p3!nLZ4s0aPjA7pGdWxOgrjTLXNHw7jk61rge1vF7GbZjIYvd6yZp9vi0UsH5KkbK-KftW5u4tH6MT8o3HvNA$)

In your revision ensure you cite all your sources (including your own works), and quote or rephrase any duplicated text outside the methods section. Further consideration is dependent on these concerns being addressed.

**We now cite these 2 published abstracts from conference presentations. We have also rephrased duplicated text that occurs outside the methods section. Duplicate text within the methods section has been mostly unrevised.**

3. We note that you have indicated that data from this study are available upon request. PLOS only allows data to be available upon request if there are legal or ethical restrictions on sharing data publicly. For more information on unacceptable data access restrictions, please see [http://journals.plos.org/plosone/s/data-availability#loc-unacceptable-data-access-restrictions](https://urldefense.com/v3/__http:/journals.plos.org/plosone/s/data-availability*loc-unacceptable-data-access-restrictions__;Iw!!Cn_UX_p3!nLZ4s0aPjA7pGdWxOgrjTLXNHw7jk61rge1vF7GbZjIYvd6yZp9vi0UsH5KkbK-KftW5u4tH6MR7IHBDDg$).

We will update your Data Availability statement on your behalf to reflect the information you provide.

**Immediately below is a link to the data repository. Thank you for updating our data availability statement on your behalf.**

[https://osf.io/6hbn7/](https://urldefense.com/v3/__https:/osf.io/6hbn7/__;!!Cn_UX_p3!nXivhQ9pAMIb2dUCbLzSKMqO6ld3e4oSB9hMmQ8jAZoIu1z7v6xS9rOYrv9uUScQRLspkMO-ZUdgSVMXe5Imh4GjyvBlS2c$)

4. Please ensure that you include a title page within your main document. You should list all authors and all affiliations as per our author instructions and clearly indicate the corresponding author.

**The title page has been added to the main document.**

5. Please amend your authorship list in your manuscript file to include authors Julie Wagner, Angela Bermudez-Millan, Richard Feinn, Rachel Lampert, Rafael Perez-Escamilla and Sofia Segura-Perez.

**These individuals are now included in the authorship list on the title page.**

6. Please include a separate caption for each figure in your manuscript.

**We now include a figure caption.**

7. Please include your tables as part of your main manuscript and remove the individual files. Please note that supplementary tables (should remain/ be uploaded) as separate "supporting information" files.

**Tables are now included in the main manuscript. We have no supplementary files.**

Reviewers' comments:

**Comments to the Author**

1. Is the manuscript technically sound, and do the data support the conclusions?

The manuscript must describe a technically sound piece of scientific research with data that supports the conclusions. Experiments must have been conducted rigorously, with appropriate controls, replication, and sample sizes. The conclusions must be drawn appropriately based on the data presented.

Reviewer #1: Yes

Reviewer #2: Partly

2. Has the statistical analysis been performed appropriately and rigorously?

Reviewer #1: Yes

Reviewer #2: Yes

3. Have the authors made all data underlying the findings in their manuscript fully available?

The [PLOS Data policy](https://urldefense.com/v3/__http:/www.plosone.org/static/policies.action*sharing__;Iw!!Cn_UX_p3!nLZ4s0aPjA7pGdWxOgrjTLXNHw7jk61rge1vF7GbZjIYvd6yZp9vi0UsH5KkbK-KftW5u4tH6MTaqPFW2g$) requires authors to make all data underlying the findings described in their manuscript fully available without restriction, with rare exception (please refer to the Data Availability Statement in the manuscript PDF file). The data should be provided as part of the manuscript or its supporting information, or deposited to a public repository. For example, in addition to summary statistics, the data points behind means, medians and variance measures should be available. If there are restrictions on publicly sharing data—e.g. participant privacy or use of data from a third party—those must be specified.

Reviewer #1: Yes

Reviewer #2: No

**Here is a link to the data repository:**

[**https://osf.io/6hbn7/**](https://urldefense.com/v3/__https:/osf.io/6hbn7/__;!!Cn_UX_p3!nXivhQ9pAMIb2dUCbLzSKMqO6ld3e4oSB9hMmQ8jAZoIu1z7v6xS9rOYrv9uUScQRLspkMO-ZUdgSVMXe5Imh4GjyvBlS2c$)

4. Is the manuscript presented in an intelligible fashion and written in standard English?

PLOS ONE does not copyedit accepted manuscripts, so the language in submitted articles must be clear, correct, and unambiguous. Any typographical or grammatical errors should be corrected at revision, so please note any specific errors here.

Reviewer #1: Yes

Reviewer #2: Yes

Reviewer #1 Comments:

The study examines household food insecurity (HFI) and autonomic nervous system (ANS) function in a subset of low-income Latinos with type 2 diabetes. The topic is relevant however, the authors have to address these comments before granting any publication consideration.
1) The authors should incorporate the summary of their policy implication(s) into the abstract.

**The last sentence of the abstract incorporates policy implications.**

2) The authors should make clear the contribution(s) of the study.

**The discussion section now makes clear the contributions of the study.**

3) The authors should compare their findings presented in the results section with previous studies and where possible give reasons to support their outcome (example, explain why (1) there was no significant difference between FI groups on A1c, blood glucose, or insulin use; (2) there was no significant difference between FI groups on education, income, or employment).

**This is now presented in the Limitations section.**

4) The authors should incorporate their findings into the conclusion section.

**The findings are incorporated into the conclusion.**

Reviewer #2: Reviewer’s comments

The study investigated the nexus between household food insecurity (HFI) and autonomic nervous system (ANS) function in a subset of low-income Latinos with type 2 diabetes with data from a stress management trial. Generally, the article is well-written. However, the authors need to improve it. Below are my suggestions and comments.

Abstract
Methods: Present the methods adopted in sentences; avoid the use of symbol where necessary for easy understanding by the readers.
Results: Present the results in sentences; avoid the use of symbol where necessary for easy understanding by the multidisciplinary readers.

**We now report methods and results in sentences without the use of symbols or statistical abbreviations.**

Introduction
The introduction is well-written. However, the authors can improve this section by clearly showing the research gap and how their study contributes to literature. In addition, they should include how the study is important to justify the current study.

**We now state the knowledge gap and how our study addresses it.**

Paragraph 2: define FI in the first use.

**We define FI in the first use (in the first paragraph).**

Results
The sentence “A total of 121 individuals had baseline assessments for the PARENT STUDY trial, 182 n=107 were randomized and n=96 completed post assessments. Of them, n = 11 did not wear 183 a Holter monitor, HRV data from n = 15 required >20% .20% interpolation and so were 184 excluded, and 1 individual did not provide food security data. Of the remaining 69, n=34 185 declined the substudy or were excluded due to hand dysfunction (e.g., arthritis) that precluded 186 the handgrip test. This yielded 35 participants for analysis” would be better under methods.

**We have moved this to the methods section.**

Discussion
The authors need to further discuss their findings by clearly given implications of each finding and link them with earlier studies that are in line or against their findings.

**We have added this to the end of the first paragraph of the discussion.**

Conclusion
The authors need to have few sentences to conclude the paper in addition to what they have in this section. What the authors currently have under this section is recommendation, which should come after drawing inferences from the study.

**We have added a sentence with a clear, concise conclusion.**
